# Supplementary material for: Normalizing and denoising protein expression data from droplet-based single cell profiling
Source: Nat Commun. 2022 Apr 19;13:2099. doi: 10.1038/s41467-022-29356-8 (PMC9018908; doi:10.1038/s41467-022-29356-8)
Supplement: Supplementary file 1 — Supplementary Information [file 41467_2022_29356_MOESM1_ESM.pdf]

Supplementary Materials

Normalizing and denoising protein expression data from droplet-based single cell profiling

Matthew P. Mulè[1,3,4] Andrew J. Martins[1,4] and John S. Tsang[1,2]
1. Multiscale Systems Biology Section, Laboratory of Immune System Biology, National Institute of Allergy and Infectious Diseases (NIAID), National Institutes of Health (NIH)
2. NIH Center for Human Immunology (CHI), National Institutes of Health (NIH)
3. NIH-Oxford-Cambridge Scholars Program, Department of Medicine, Cambridge University
4. These authors contributed equally Matthew P. Mulè, Andrew J. Martins
Correspondence to: john.tsang@nih.gov

Contents:

Supplementary Note p.2

Supplementary Figures p.8

The method presented in this paper is available as an open source R package “dsb” available on CRAN. For up to date tutorials, please see the package documentation and vignettes:

https://CRAN.R-project.org/package=dsb

Supplementary Note

Robustness of protein-specific noise estimation assessed by using different approaches to define empty/background droplets

The dsb package utilizes the raw (unfiltered) output of UMI count aligners such as Cell Ranger, Kallisto[1] or as we used here, CITE-seq Count[2]. The unfiltered output (for example, in Cell Ranger, the *raw* output) of droplet barcodes versus UMI counts includes all cell containing and empty (or “background”) droplets, both of which can be inferred using thresholding methods based on the mRNA and protein library sizes in combination with algorithms like EmptyDrops[3] to distinguish cells from background noise (as done by default by Cell Ranger)–see dsb package documentation tutorial. In all datasets analyzed by us to date, a considerable number (at least 50,000 after QC) of background droplets (i.e., barcodes inferred to not contain at least one cell) can be found using library size based thresholding (see below for robustness assessments). The protein counts derived from these background droplets reflect contributions from ambient antibodies, which as shown in the main text, were highly correlated with the protein counts detected in unstained control cells included in our experiment. Thus, as discussed in the main text, protein counts in empty droplets can serve as an estimate of the expected ambient levels of antibodies. To assess the robustness of estimating protein-specific noise in relation to how background droplets are defined, we compared three approaches to define background droplets. As detailed in our previous report[4], due to the number of samples included in our experiment, demultiplexing samples required data from both sample barcode (“cell hashing”) antibodies and mRNA (for genetic based demultiplexing, i.e., by cross referencing independently generated patient genotype data using demuxlet, see Methods and Kotliarov *et. al.* 2020). After removing doublets and defining singlets on the basis of data from both the hashing antibodies and genotypes, the remaining (non-doublet, non-singlet) droplets were used to define background droplets in two different ways. First, "Library size background droplets" were defined solely based on library size information where we used clear breaks in the distribution of protein library sizes across the remaining droplets followed by removal of droplets in the top 10th percentile based on the mRNA library size in order to eliminate droplets containing low quality cells. The library size approach to define background droplets is most compatible with experiments that do not have sample multiplexing or hashing antibody data, such as the external CITE-seq datasets from 10X Genomics used in this paper (Fig. 3 and Supplementary Fig. 4).

The second background droplet inference method we tested requires CITE-seq experimental workflows similar to ours, where many samples are multiplexed in the same experiment using sample barcoding antibodies (and/or genetic based demultiplexing). After using Seurat's K-medoids function to computationally classify cell barcodes as containing singlets, doublets, or negatives based on the hashing antibody counts, we defined "Hashing background droplets" as those classified as "negative" by this demultiplexing software. These droplets had staining below the threshold to be called positive for any one of the hashing antibodies and

therefore in principle, their antibody counts should reflect only ambient capture. Such hashing "negatives" were an order of magnitude fewer in number than those determined by library size above, largely due to the threshold used for determining whether a droplet is included in the hash demultiplexing pipeline (the top 35,000 barcodes from each lane). Hashing background droplets were further filtered to: 1) include only droplets classified as "ambiguous" by SNP demultiplexing (via demuxlet), i.e., these cannot be attributed to a single or multiple distinct donors based on cross-referencing mRNA reads in the droplet with independently generated genotype data, and 2) exclude any droplet with >80 unique mRNAs to remove cell-containing droplets with low-quality mRNA capture. Using this alternative method to define background droplets, we similarly observed that the relative amount of antibody was highly correlated between unstained cells and these background droplets (along the unity line in Supplementary Fig. 1b, top).

Interestingly, while the correlation was similarly high, antibody levels in unstained cells or in hashing background droplets were greater than those in library size background droplets (Supplementary Fig. 1b top vs bottom). The greater magnitude of antibody counts by a multiplicative factor in log-count space (slope in bottom panel of Supplementary Fig. 1b is 1.24 with near zero intercept) suggests that unstained cells and demultiplexing background droplets capture additional antibodies. Unstained cells may serve as an additional antibody capturing "reservoir", e.g., due to non-specific (or specific) binding of the ambient antibody remaining after multiple wash steps. However, this would not explain their concordance with demultiplexing background, which, as supported by both genetic (via demuxlet classification) and barcoding antibody (via Seurat k-medoids classification) data, should have a low chance of containing fully intact cells. It is still possible, despite filtering out droplets with low mRNA counts, that demultiplexing background droplets contained some very low-quality cells or cell membrane debris that together could capture additional antibodies from the environment via specific/non-specific binding. Demultiplexing background droplets could also have more ambient mRNA (as described above in order to be included in the hashing antibody demultiplexing step) than droplets defined using the protein library size distribution alone, and thus they (as also in the unstained control cell droplets) could conceivably serve as an additional set of free antibody-capturing molecules. Importantly, however, we emphasize the difference between empty/background droplets defined using protein library size distribution versus hashing antibody demultiplexing had negligible effect in the resulting dsb normalized values (see below).

We further investigated a third approach to estimate protein background noise–the mean of each protein across the subset of *stained cells* that were inferred to belong to the "*negative"* population for each protein. Without dsb rescaling, we fit a two component Gaussian mixture model to the log + 1 transformed count of each protein across single cells, resulting in 2 populations of cells: those positive or negative for the protein. Each protein's background mean, "*A*" (see Supplementary Fig.1a), reflects the average log transformed count of the non-staining cell population for that protein, i.e., cells that do not express that protein. The protein level in

unstained controls and empty drops were both highly correlated with *A* (Supplementary Fig. 1c). Thus, antibody levels in unstained droplets on average are similar to those in droplets with stained cells not expressing the target protein.

We thus have tested three different ways to estimate the average background protein noise correlated across droplets. We further found that the noise signal captured in library size background droplets appears to be universally found in data from all of the droplet-based oligo barcoded antibody experiments we examined and is thus a generalizable method of estimating noise.

Importance of using isotype control antibodies for estimating cell-intrinsic normalization factors

Our method is compatible with experiments lacking isotype controls by either not removing the cell-specific technical variation (use *denoise.counts = FALSE* in dsb*)* or by removing the technical component with a single fitted parameter, the per-cell mean of the background protein population (parameters *denoise.counts = TRUE, use.isotype.control = FALSE*). However, additional analyses further support our findings that inclusion of isotype controls benefits cell to cell technical noise correction (step II). Despite the ability of $\mu1$ alone to provide information about the cell-intrinsic technical component, we recommend the inclusion of multiple isotype controls in CITE-seq experiments to serve as anchors for better estimation of technical normalization factors because $\mu1$ alone may carry signals beyond those from technical factors (e.g., low-level antigen specific binding). In our data for example, $\mu1$ exhibited greater correlation with $\mu2$ than did the mean of the isotype controls (Supplementary Figs. 3b,c), including when sub-sampling random draws of four proteins from those used to compute $\mu1$ within each cell to assess whether signal from four background proteins is equivalent to that of four isotype controls (Supplementary Fig. 3d). Furthermore, even with isotype controls as anchors, the estimated cell-intrinsic background may encompass signals from non-specific binding to surface Fc receptors. Cell types such as monocytes with higher relative Fc receptor expression may thus receive more correction than other cell types. However, empirically we have not found this to have adverse effects on normalized values in populations such as monocytes, cell type identification, or downstream analysis (Supplementary Figs. 6b, c). Careful blocking of Fc receptors before antibody staining, which is standard practice and was performed in our experiments, likely contributed to mitigating this effect.

Robustness of dsb normalized values to background droplet definition

Given the strong correlation observed between average protein levels in unstained control cells and both empty drops and droplets with stained cells expressing background level of the protein (Supplementary Fig. 1b,c), ambient antibodies appear to capture the major noise component that

contributes to each protein's specific noise floor. In external 10X Genomics CITE-seq datasets, we distinguished between empty droplets and cells using the Cell Ranger alignment tool which uses a method inspired by the EmptyDrops[3] algorithm to identify cell-containing droplets. The number of estimated cell-containing droplets depends on the number of cells loaded during droplet generation which should then inform the value input to the Cell Ranger *expect_cells* parameter; typical experiments recover on the order of 10^3 to10^4 droplets. Empty droplets capturing ambient ADTs, typically between $5x10^4$ to $1x10^5$ in number, can be robustly defined from the remaining, non-cell-containing barcodes in the raw output matrix. The raw output matrix lists all possible cell barcode combinations (more than 6 million barcodes in the Version 3 and Next Gem assays), many of which have no evidence of capture in the experiment (i.e. no data for mRNA or ADT reads) and empty droplets capturing ambient ADT must be subset from this output in order to avoid biasing the background estimates. The steps to complete this process are completed in a few lines of code as detailed in the dsb package documentation. A substantial subset of the cell barcodes estimated by Cell Ranger to not contain a cell had ADT reads with an order of magnitude lower protein library size compared to the cell-containing droplets. We then applied quality-control thresholds determined based on protein and mRNA counts for each dataset, for example, excluding certain "empty" droplets from being used in the background distribution that likely corresponded to potentially low-quality cells (e.g., removing empty droplets with more than 80 unique mRNA). This procedure revealed a clear population of more than 50,000 background droplets in each dataset. In some external datasets, there were two distinct background populations based on protein library size (Supplementary Fig. 7a). dsb normalized values were robust to using different background subpopulations (Supplementary Fig. 7a,b). When only the lower ADT background peak was used to simulate an experiment with extremely low background, dsb normalized values still separated canonical cell populations but were less zero-centered due to the low estimated background for some proteins (third row, Supplementary Fig. 7b). We have not encountered a dataset like this simulation scenario to date, however, in the future as antibody panels continue to increase in size, some antibodies may be titrated down to extremely low concentrations. Theoretically, this could decrease background levels in empty droplets for certain proteins to a level that could impact the first step of dsb as shown above. Our method could be easily adjusted in this hypothetical case by modifying the standardization step to accommodate lower background dispersion.

Within batch normalization vs. pooled normalization across multiple batches

The experimental design of the main dataset used here to develop our approach include n=20 unique donors distributed over two experimental batches; this presented multiple options for dsb normalization. Background/empty drops could be defined with either of the two methods described above (demultiplexing or library size), and cells could then be normalized by combining all cells / background into a single matrix and normalizing both batches together, or each batch of cells could be normalized separately, using only the empty droplets within each batch. To test how

robust the resulting dsb normalized values were to single vs multi-batch normalization, as well as to further validate the findings described above on the robustness of dsb normalized values to different definitions of background, we tested the 4 possible normalization schemes with background droplets classified by either protein library size distribution or demultiplexing, then normalized with dsb by either merging cells and background from both batches together, or normalizing each batch separately. The resulting dsb normalized values were consistently similar across all four of these normalization schemes (Supplementary Fig. 8). Since we expect ambient antibody to be a major contributor of correlated noise across cells, experimental standardization of staining time and the number of washing steps prior to droplet generation as well as use of the same pool of manually concentrated antibody on each batch could be important contributing factors in mitigating batch to batch variations. Our method is not designed as a batch effect removal tool, however, as enabled by the standard, normalized expression value scale from dsb, the approach of applying a uniform background cutoff threshold across proteins in diverse datasets can potentially help mitigate batch effects. The performance of existing batch correction tools[5,6] including single cell integration methods[7–10] on ADT data could be an area of further investigation to compare upstream dsb to other normalization methods as more datasets become available.

References

1. Melsted, P. et al. Modular, efficient and constant-memory single-cell RNA-seq preprocessing. *Nat. Biotechnol.* **39** 813–818 (2021).
2. Roelli, P., bbimber, Flynn, B., santiagorevale & Gui, G. Hoohm/CITE-seq-Count: 1.4.2. (2019). doi:10.5281/ZENODO.2590196
3. Lun, A. T. L. *et al.* EmptyDrops: Distinguishing cells from empty droplets in droplet-based single-cell RNA sequencing data. *Genome Biol.* **20**, 1–9 (2019).
4. Kotliarov, Y. et al. Broad immune activation underlies shared set point signatures for vaccine responsiveness in healthy individuals and disease activity in patients with lupus. *Nat. Med.* **26**, 618–629 (2020).
5. Ritchie, M. E. *et al.* limma powers differential expression analyses for RNA-sequencing and microarray studies. *Nucleic Acids Res.* **43**, e47 (2015).
6. Johnson, W. E., Li, C. & Rabinovic, A. Adjusting batch effects in microarray expression data using empirical Bayes methods. *Biostatistics* **8**, 118–127 (2007).
7. Stuart, T. *et al.* Comprehensive Integration of Single-Cell Data. *Cell* **177**, 1888-1902.e21 (2019).
8. Welch, J. D. *et al.* Single-Cell Multi-omic Integration Compares and Contrasts Features of Brain Cell Identity. *Cell* **177**, 1873-1887.e17 (2019).
9. Korsunsky, I. *et al.* Fast, sensitive and accurate integration of single-cell data with Harmony. *Nat. Methods* **16**, 1289–1296 (2019).
10. Gayoso, A. et al. Joint probabilistic modeling of single-cell multi-omic data with totalVI. *Nat. Methods.* 18, 272–282 (2021).

Supplementary Figures

Supplementary Fig. 1. Robustness assessment of estimating ambient ADT noise in cell-containing droplets using ADT levels in empty droplets via comparison with unstained controls.

Supplementary Fig. 2. Robustness assessment of models fitted to each cell in dsb (step II part I).

Supplementary Fig. 3. Analysis of isotype control contribution to dsb technical component and comparison of dsb normalized values to centered log ratio normalization.

Supplementary Fig. 4. Analysis of dsb normalization on external CITE-seq datasets.

Supplementary Fig. 5. Analysis of dsb normalization on TEA-seq, ASAP-seq and Mission Bio datasets.

Supplementary Fig. 6. Manual and automatic cell type identification with protein levels after dsb normalization from healthy donor PBMC data (data from Kotliarov *et. al.* 2020).

Supplementary Fig. 7. Robustness assessment of dsb normalized values to different subsets of empty droplets used for background correction with dsb.

Supplementary Fig. 8. Batch processing with dsb: analysis of merging multiple batches then normalizing, vs. separate normalization applied within each batch.

Supplementary Fig. 9. Additional figures from analysis of TEA-seq data (data from Swanson *et. al.* 2021).

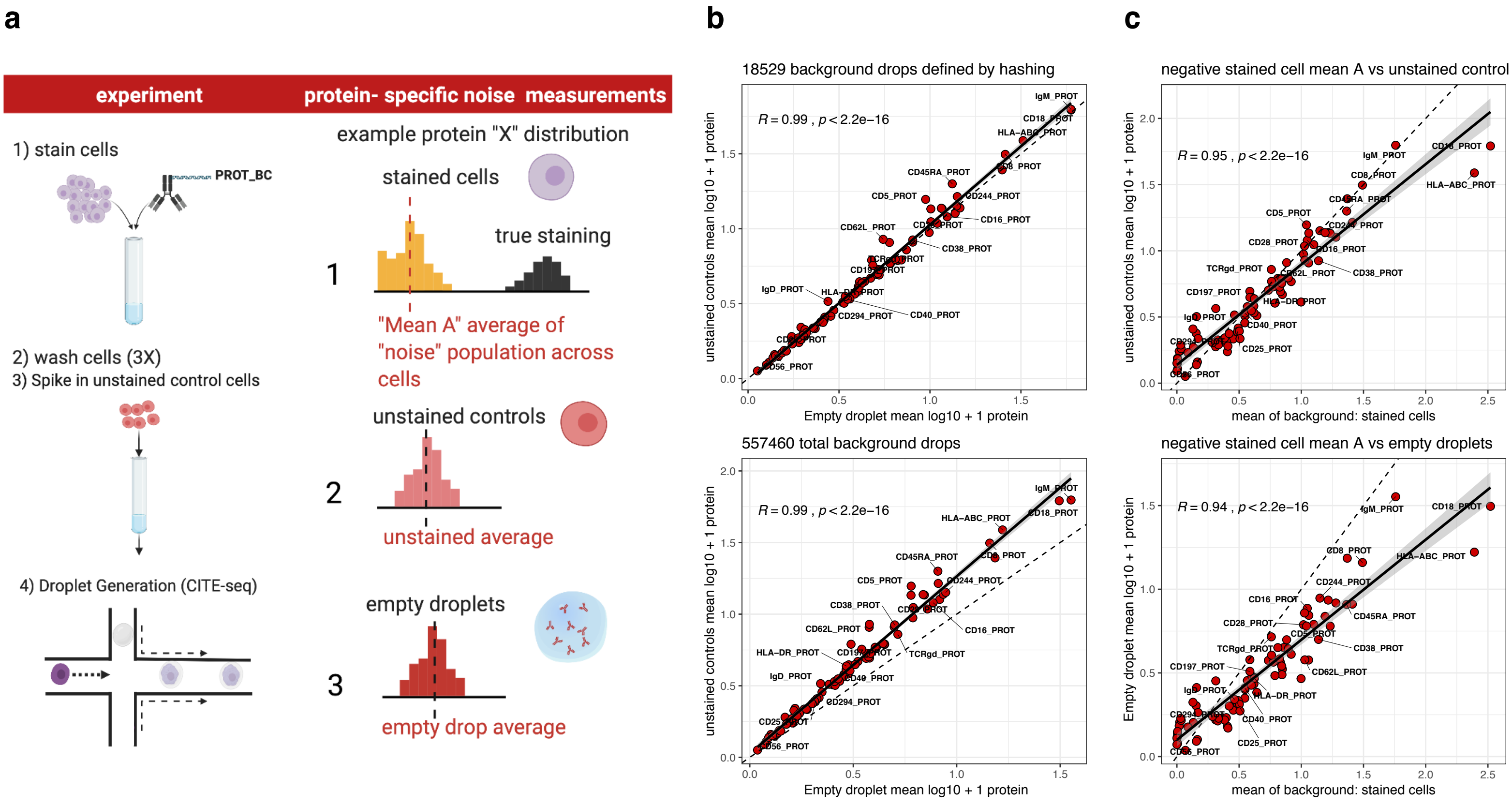
a
experiment
protein- specific noise measurements
1) stain cells
PROT_BC
2) wash cells (3X)
3) Spike in unstained control cells
4) Droplet Generation (CITE-seq)
example protein "X" distribution
stained cells
true staining
1
"Mean A" average of "noise" population across cells
unstained controls
2
unstained average
empty droplets
3
empty drop average
b
18529 background drops defined by hashing
R = 0.99 , p < 2.2e−16
unstained controls mean log10 + 1 protein
Empty droplet mean log10 + 1 protein
557460 total background drops
R = 0.99 , p < 2.2e−16
c
negative stained cell mean A vs unstained control
R = 0.95 , p < 2.2e−16
mean of background: stained cells
negative stained cell mean A vs empty droplets
R = 0.94 , p < 2.2e−16
Empty droplet mean log10 + 1 protein

Supplementary Figure 1
a. Expanded from Fig. 1a: to assess the relative contribution of the ambient antibody component of noise correlated across droplets, three different measurements of protein-specific background noise were defined for each protein: 1) (top row, right column) for each protein, the average log transformed value of the subset of stained cells that were not part of the proteins “positive” population and comprised the “non-staining” population of cells (the negative cell population for each protein was inferred through a Gaussian mixture model fit separately to each protein, see Methods) 2) (middle row, right column): unstained control cells spiked into the cell mixture prior to droplet generation as shown in the experiment diagram (left column), 3) (bottom row, right column): empty droplets as defined by either the protein library size distribution or inferred by sample barcode antibody demultiplexing (see Methods). **b.** Pearson correlation coefficient and p value (two sided) between unstained control cells (y-axis) and empty droplets (x-axis) with empty droplets defined by either demultiplexing (top “hashing background droplets”) or library size distribution (bottom, “library size background droplets”, see supplemental note) **c.** Pearson correlation coefficient and p value (two sided) between y-axis: unstained controls (top panel) or library size background droplets (bottom panel) versus x-axis: the mean of the protein in stained cells that were negative for the protein (“mean A” as shown in top panel of a). In all plots the dashed line at unity (y = x) is shown for reference and the solid line is the fitted regression line with the shaded region representing the 95% confidence interval of the linear model fit centered around the fitted values. Illustration created with BioRender.com.

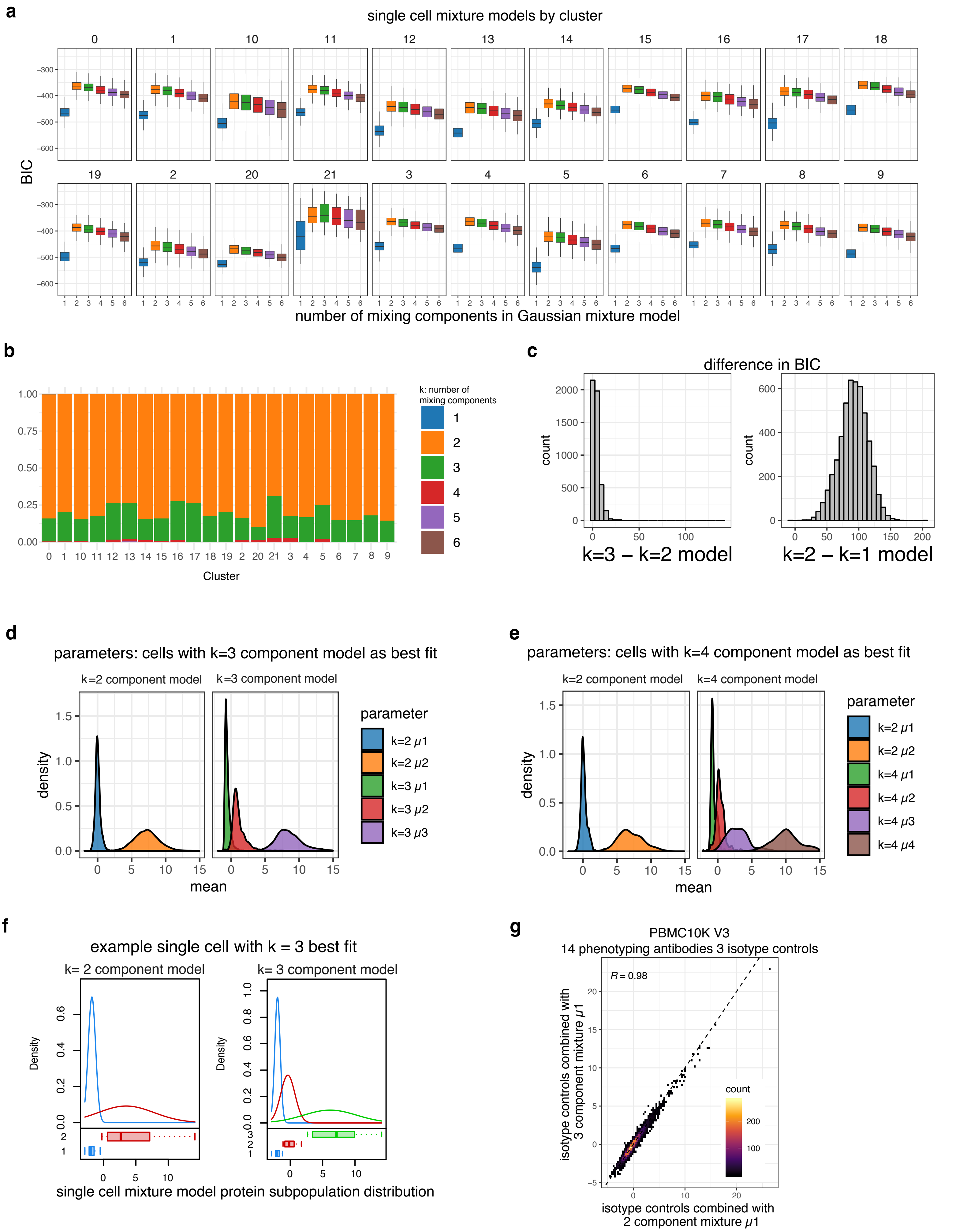
a
single cell mixture models by cluster
BIC
number of mixing components in Gaussian mixture model
b
k: number of mixing components
Cluster
c
difference in BIC
count
k=3 – k=2 model
k=2 – k=1 model
d
parameters: cells with k=3 component model as best fit
k=2 component model
k=3 component model
density
mean
parameter
k=2 μ1
k=2 μ2
k=3 μ1
k=3 μ2
k=3 μ3
e
parameters: cells with k=4 component model as best fit
k=2 component model
k=4 component model
density
mean
parameter
k=2 μ1
k=2 μ2
k=4 μ1
k=4 μ2
k=4 μ3
k=4 μ4
f
example single cell with k = 3 best fit
k= 2 component model
k= 3 component model
Density
single cell mixture model protein subpopulation distribution
g
PBMC10K V3
14 phenotyping antibodies 3 isotype controls
R = 0.98
count
isotype controls combined with 3 component mixture μ1
isotype controls combined with 2 component mixture μ1

Supplementary Figure 2

Assessment of the modeling assumptions for defining each cell's background protein population mean $\mu 1$ with a k=2 component mixture model for use in the per-cell technical component regressed out of dsb normalized counts in step II–see related figures on external validation datasets (Supplementary Fig. 4). **a.** Gaussian mixture model fits (from Figs. 1d-e) partitioned by each protein-based cell cluster (clusters are the same as defined after dsb normalization in Kotliarov *et. al.* 2020). Boxplots show the median BIC with hinges at the 25th and 75th percentile and whiskers extending plus or minus 1.5 times the inter quartile range. The number of cells for each cluster: cluster 0 = 10927, 1 = 8268, 10 = 1250, 11 = 967, 12 = 853, 13 = 773, 14 = 371, 15 = 343, 16 = 292, 17 = 225, 18 = 218, 19 = 165, 2 = 6655, 20 = 137, 21 = 74, 3 = 4853, 4 = 4507, 5 = 4236, 6 = 2510, 7 = 2287, 8 = 1892, 9 = 1398. **b.** Similar to the barplot shown in Fig.1e, but partitioned by high resolution protein based cluster; cells with k = 3 as the best fit were not biased to a specific protein-based cluster. **c.** For 17% of cells with k = 3 models having the best fit (cells from Fig 1e), the difference in BIC between k = 3 vs. k = 2 and k = 2 vs. k = 1 models is shown. **d.** The distribution of Gaussian mixture model subpopulation means for k = 2 and k = 3 models for the subset of cells with k = 3 as the optimal fit (means < 15 shown to focus on µ1 distributions) shows k = 3 and k = 2 models fit similar values for µ1 in these cells. **e.** As in (d); the small minority of cells (shown in red in (b)) with k = 4 as the best fit. **f.** A single arbitrary example cell that had an optimal BIC with the k = 3 model; the distribution of inferred mixture model means is shown for the 2-subpopulation (left) and 3-subpopulation (right) model fits showing overlapping value for $\mu 1$. **g**. As in Fig.1h, using the 10X Genomics CITE-seq dataset "PBMC V3 10K" which measured only 14 surface phenotyping proteins and 3 isotype controls. The distribution of the dsb technical component as calculated using a 2 component (x-axis) vs. 3 component (y-axis) mixture model to define the µ1 parameter.

Supplementary Fig. 3

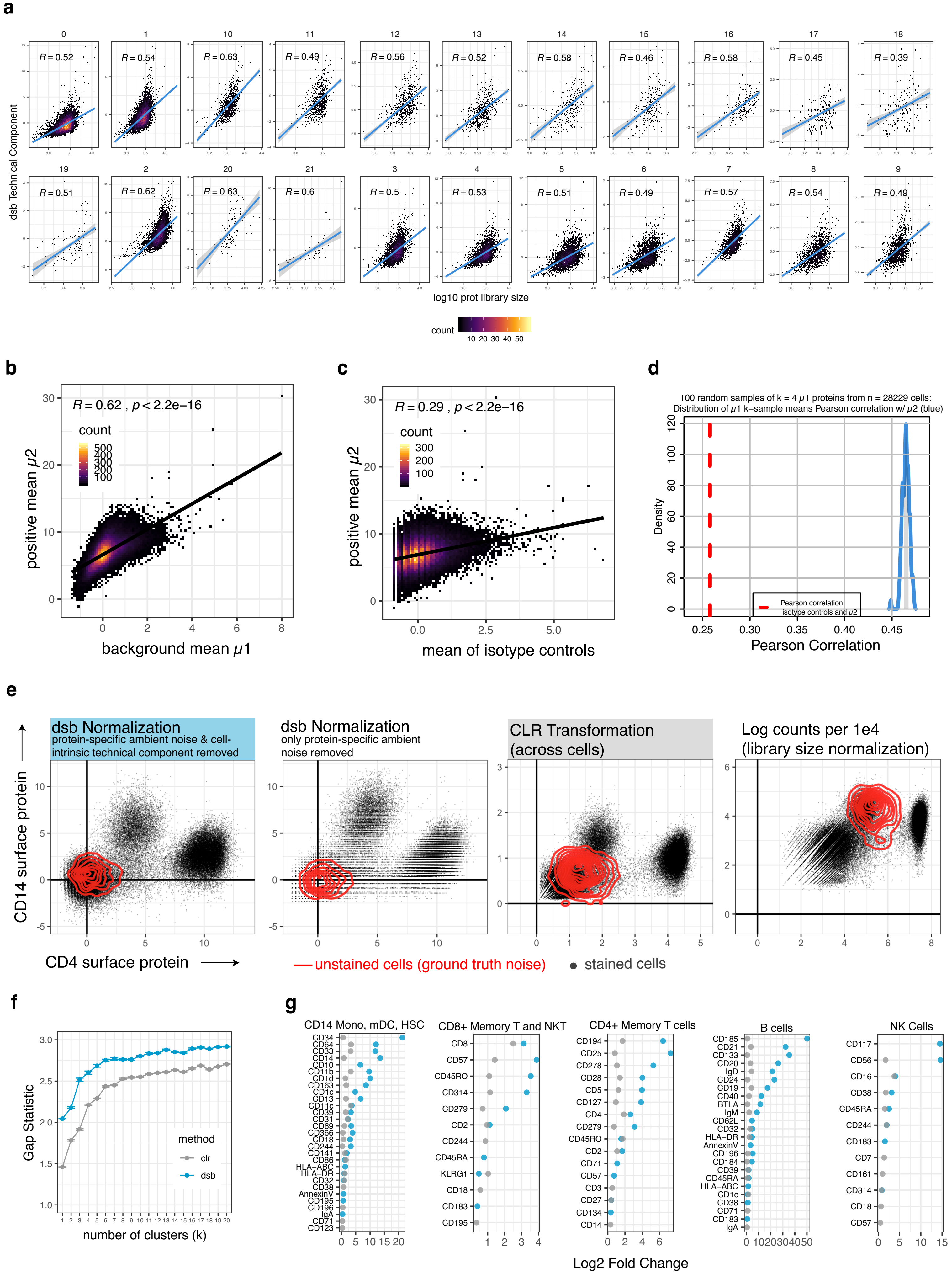

Supplementary Figure 3

a. Each cell's inferred technical component λ (y-axis) vs the cell's protein library size; panel number indicates protein-based clusters (see Methods) as shown in Supplementary Fig. 6 and Fig 3. R indicates Pearson correlation coefficient of linear fit, 95% confidence interval highlighted in grey. **b.** The Pearson correlation coefficient and p value (two sided) between $\mu 1$ and $\mu 2$ from single cell k = 2 component mixture models fit across all proteins in each cell. **c.** The average of isotype controls after dsb normalization step I (ambient correction) vs $\mu 2$ as in (b) Pearson correlation coefficient and p value (two-sided). **d**. The distribution of n=100 Pearson correlation coefficients between each cell's $\mu 2$ and 100 random samples of k=4 $\mu 1$ proteins from each single cell (blue) shaded region is the 50% highest density interval, red line is the Pearson correlation coefficient of $\mu 2$ and the mean of isotype controls in each cell from batch 1 (28,229 cells). **e.** Single cell protein expression of CD4 vs. CD14 normalized by different methods. Contour lines in red are the distribution of CD4 and CD14 in unstained control cells after normalization in the exact same way as the stained cells in black within each panel, including dsb normalization using the same empty droplets for ambient correction of the unstained cells. Outlier cells (less than 0.3% of total cells in any panel) are removed to focus on the three main cell populations. The default implementation of dsb using steps I and II (top left panel) and CLR across cells are shaded in blue and grey respectively as these methods are further compared in subsequent panels and in Figs. 4 and 5. **f.** The Gap Statistic (see Methods) for different number of clusters (k) obtained using the k-medoids clustering algorithm on normalized protein values from dsb vs. CLR (across cells), bars are standard errors of the gap statistic calculated by the clusGap R function. **g.** Log fold-change estimates from differential expression analysis of proteins for each major cell type shown in comparison with the rest of the cell types (blue – dsb, grey – CLR across cells).

Supplementary Fig. 4

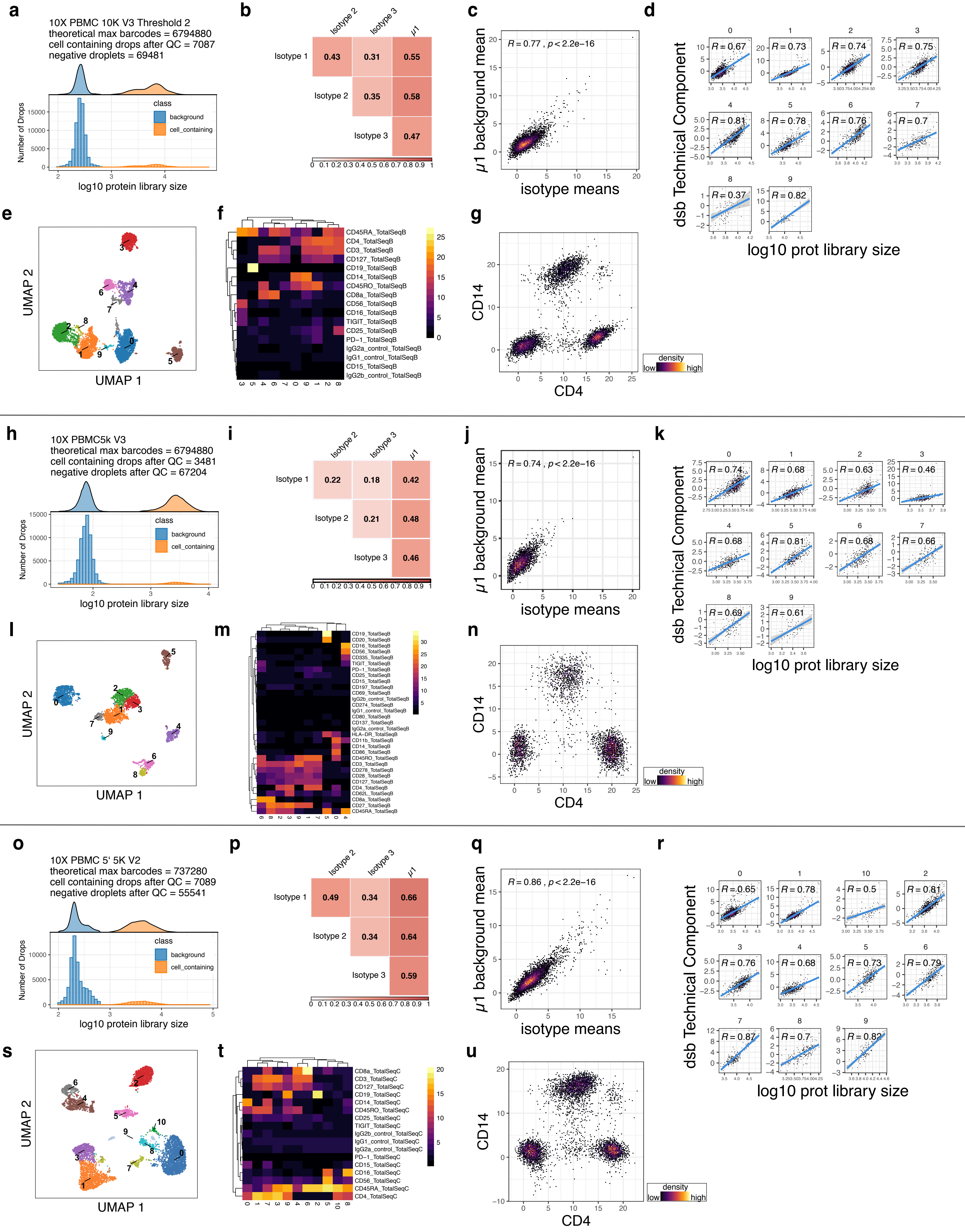

Supplementary Figure 4
Panels as shown in Fig. 2 (10X Genomics dataset "PBMC 5k" Next Gem assay) for additional 10X genomics datasets using different assays and protein panels. **a-g** "PBMC 10k" V3 assay, **h-n** "PBMC 5k" V3 assay and **o-u** "PBMC 5k" 5 prime V2 assay. 95% confidence intervals of linear model fits (d,k,r) in grey. Pearson correlation coefficients and p values (two sided) are shown (c, j, q).

Supplementary Fig. 5

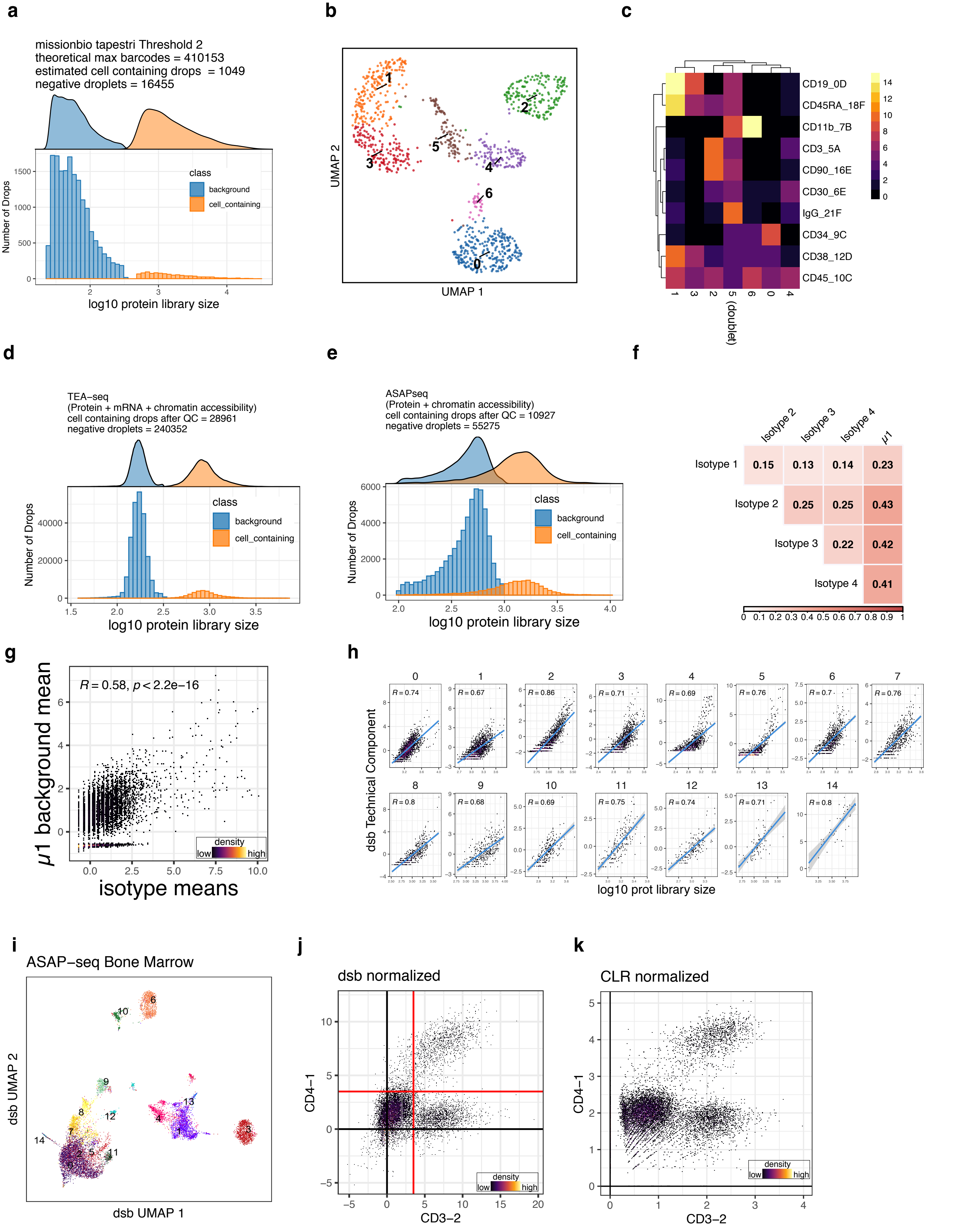

Supplementary Figure 5

a. A mixture of n=4 leukemia cell lines from example data generated via the Mission Bio 'Tapestri" platform for simultaneous "proteogenomic" assessment of surface proteins and DNA**.** The protein library size (total UMI) distribution was used to distinguish between cell-containing and empty droplets without cells. **b.** UMAP analysis based on dsb normalized values; cells are labeled by graph-based cluster identity. **c.** heatmap of the average expression of each dsb-normalized protein in each cluster. The range of values is on the same scale for all proteins, ranging from less than 0 to 14, corresponding to 14 standard deviations from the average background level estimated using empty droplets– cell-to-cell technical variations were not inferred by calculating the technical component for each cell (step II of dsb) in this dataset due to the small number of proteins profiled (n=10, see Supplementary Note and Methods). **d.** As in (a) for TEA-seq and **e.** ASAP-seq datasets. Cell-containing droplets defined by the QC pipeline from Swanson *et. al.* and Mimitau *et. al.*, respectively; note that only protein was used to estimate background from the subset of droplets that did not meet cell QC for the ASAP-seq dataset (see methods). **f.** As in Fig. 1f, correlation matrix of variables comprising the dsb technical component. **g.** As in Fig.1g, isotype control mean vs. background mean per cell. Pearson correlation coefficient and p value (two sided) is shown. **h**. As in Supplementary Fig. 3a, relationship between protein library size and the dsb technical component. Linear trend shown in blue with 95% confidence intervals in grey. **i.** UMAP projection and clusters based on dsb normalized protein values. **j.** Biaxial plot of CD3 vs. CD4 with the dsb threshold of 3.5 shown. **k.** As in (j) but with data normalized using the CLR transformation (across cells).

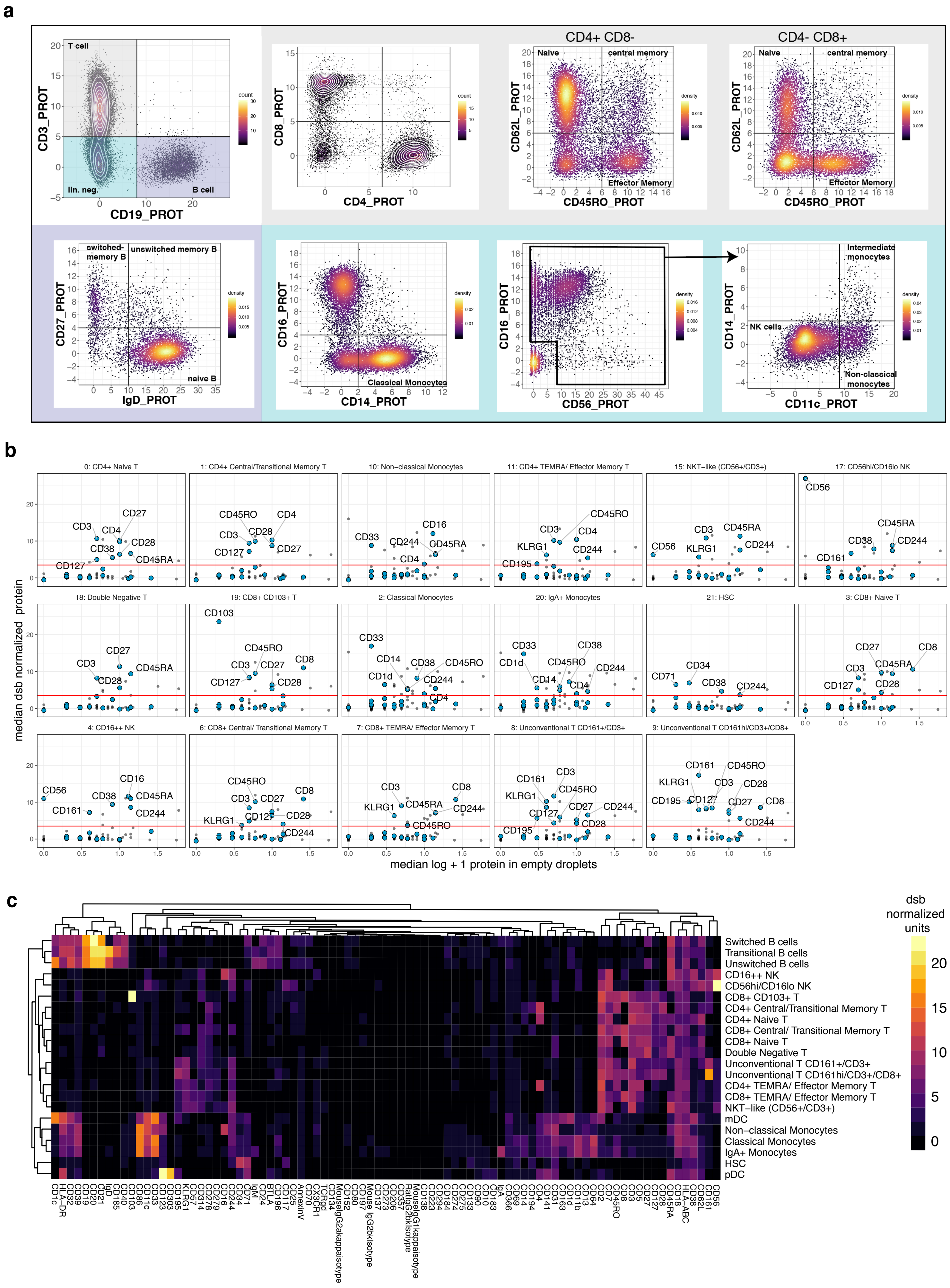
a
T cell
lin. neg.
B cell
CD3_PROT
CD19_PROT
CD8_PROT
CD4_PROT
CD4+ CD8-
CD4- CD8+
Naive
central memory
Effector Memory
CD62L_PROT
CD45RO_PROT
switched-memory B
unswitched memory B
naive B
CD27_PROT
IgD_PROT
CD16_PROT
CD14_PROT
Classical Monocytes
CD56_PROT
Intermediate monocytes
NK cells
Non-classical monocytes
CD11c_PROT
b
0: CD4+ Naive T
1: CD4+ Central/Transitional Memory T
10: Non-classical Monocytes
11: CD4+ TEMRA/ Effector Memory T
15: NKT-like (CD56+/CD3+)
17: CD56hi/CD16lo NK
18: Double Negative T
19: CD8+ CD103+ T
2: Classical Monocytes
20: IgA+ Monocytes
21: HSC
3: CD8+ Naive T
4: CD16++ NK
6: CD8+ Central/ Transitional Memory T
7: CD8+ TEMRA/ Effector Memory T
8: Unconventional T CD161+/CD3+
9: Unconventional T CD161hi/CD3+/CD8+
median dsb normalized protein
median log + 1 protein in empty droplets
c
dsb normalized units
Switched B cells
Transitional B cells
Unswitched B cells
CD16++ NK
CD56hi/CD16lo NK
CD8+ CD103+ T
CD4+ Central/Transitional Memory T
CD4+ Naive T
CD8+ Central/ Transitional Memory T
CD8+ Naive T
Double Negative T
Unconventional T CD161+/CD3+
Unconventional T CD161hi/CD3+/CD8+
CD4+ TEMRA/ Effector Memory T
CD8+ TEMRA/ Effector Memory T
NKT-like (CD56+/CD3+)
mDC
Non-classical Monocytes
Classical Monocytes
IgA+ Monocytes
HSC
pDC

Supplementary Figure 6

a. Biaxial gating strategy for identifying major immune cell subsets with dsb normalized values. Grey = T cells, Blue = Monocytes, Purple = B cells. **b.** As in Fig. 3e, the average log transformed protein count in empty droplets (x-axis) vs the average dsb normalized values (y-axis) for each protein-based cell cluster–the threshold above which proteins are annotated in the plot is 3.5 corresponding to 3.5 standard deviations above expected noise +/- the technical component correction applied in step II (see methods). In each plot the same subset of proteins is highlighted in blue for comparison of individual marker values between clusters; proteins highlighted in blue are CD1d, CD1c, CD14, CD103, CD16, CD3, CD4, CD8, CD28, CD161, CD45RO, CD45RA, CD33, CD56, CD71, CD27, CD244, KLRG1, CD195, CD38, CD127, CD16, CD34. When the protein value is above the 3.5 threshold, it is labeled with the protein name in each individual panel. **c.** Heatmap of average dsb protein normalized expression in each cluster.

Supplementary Fig. 7

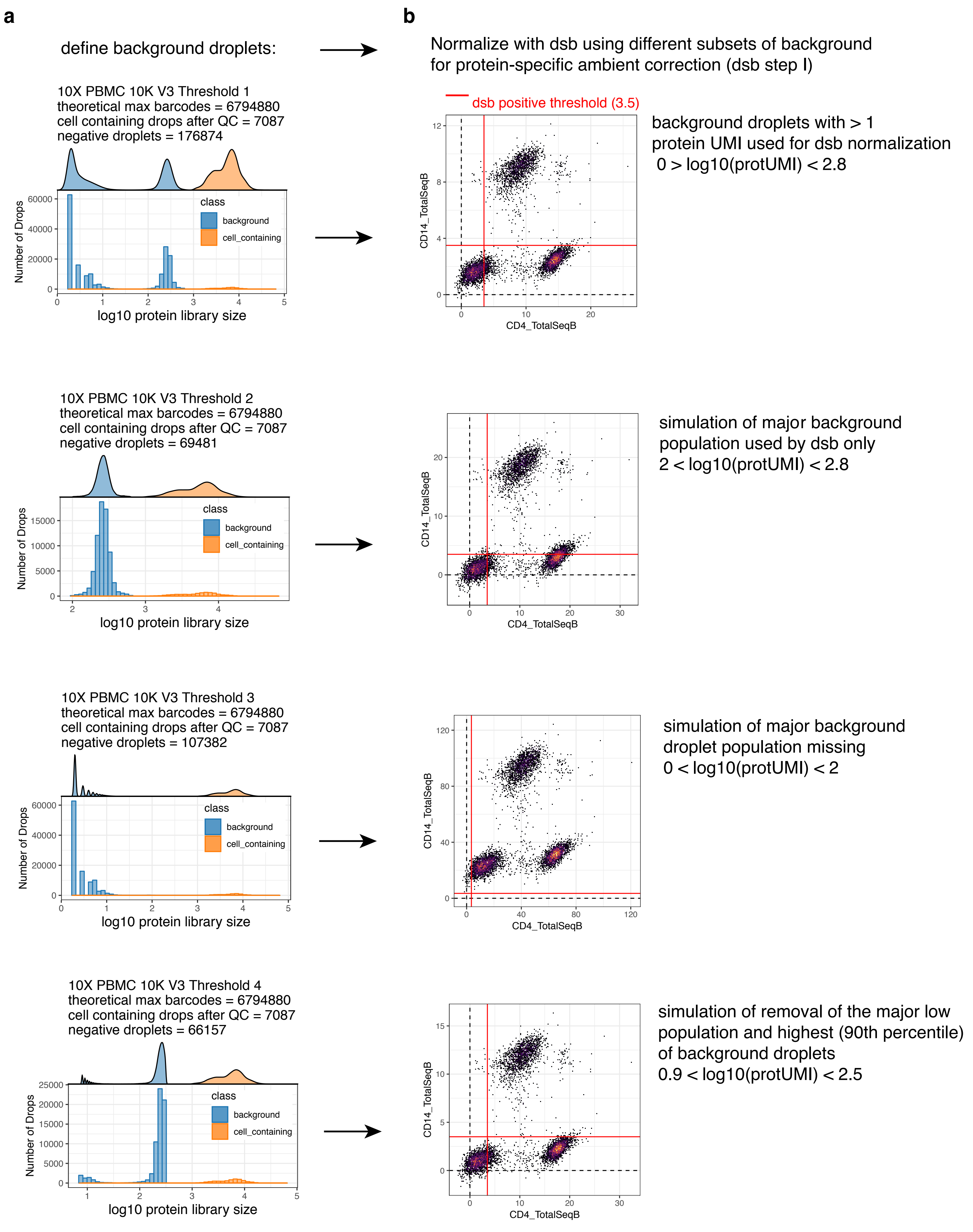

Supplementary Figure 7

Robustness of dsb normalized values to different definitions of background droplets. **a.** Distribution of protein library size for the 10X genomics Chromium Version 3 “PBMC 10K” dataset which had a bimodal distribution for the non-cell-containing droplets shown in blue. In each row, a different threshold based on the protein library size was used to define background droplets, which were then used to normalize the same population of cell-containing droplets (shown as the orange distribution) with the dsb package. **b.** The dsb normalized values are shown for canonical protein-based phenotypes with biaxial scatterplots. The scale of the 3rd row is negatively impacted by eliminating the major empty droplet background peak with greater mean value and only using the empty droplet background peak with very low mean protein library size.

Supplementary Fig. 8

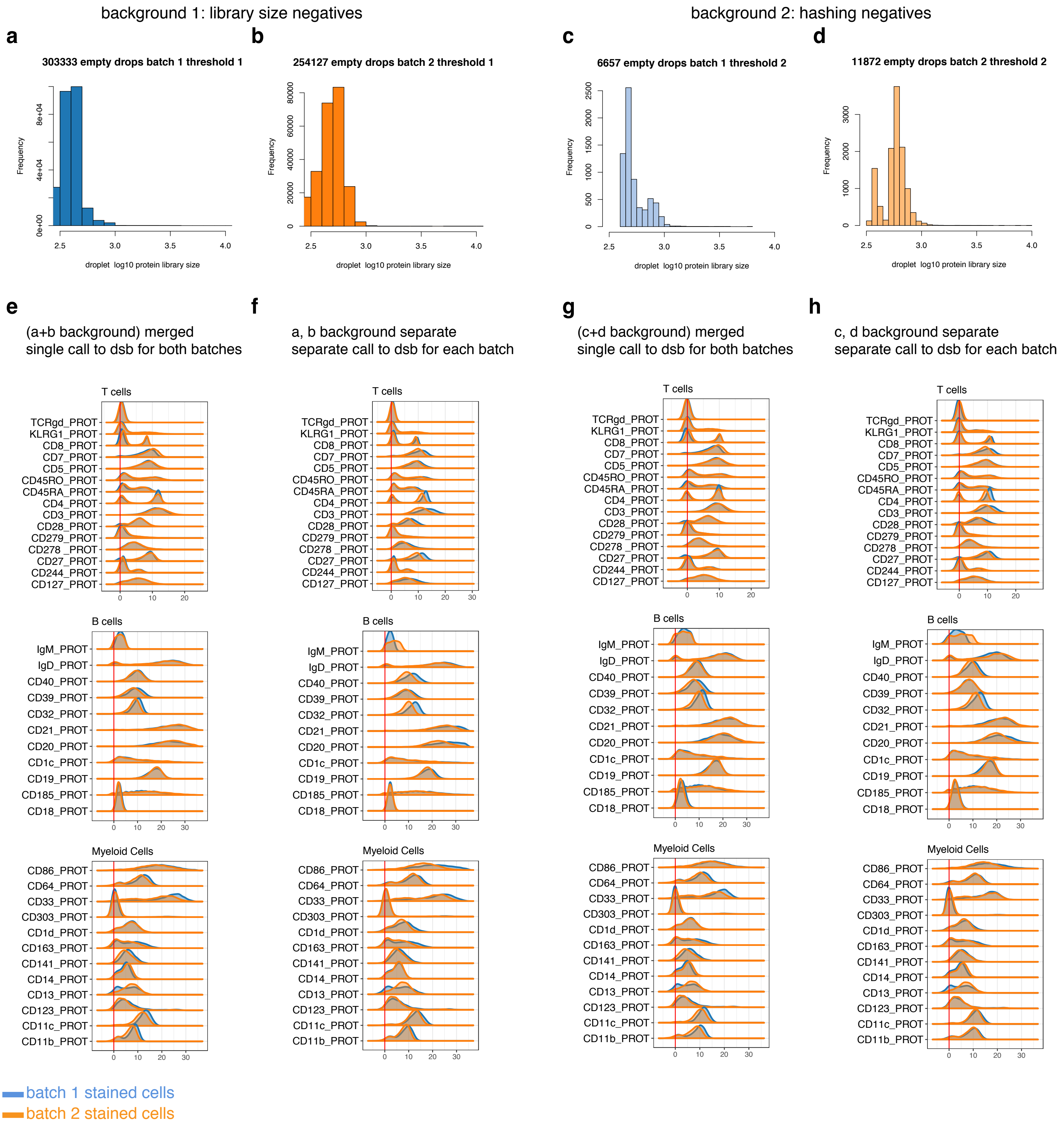

Supplementary Figure 8

Stability of dsb normalized values when processing multiple batches in a single normalization vs normalizing each batch separately, both using two definitions of background droplets with the dsb package. **a–d** show protein library size distributions of background droplets defined using either the protein library size distribution alone or droplets defined as negative during demultiplexing (see Supplemental note) across n = 2 batches. The raw Cell Ranger outputs from each staining batch of cells were split across n=6 lanes per batch of the 10X Chromium instrument and for each definition of background, the dsb results for merged vs split batch normalization are shown in **e-h**.

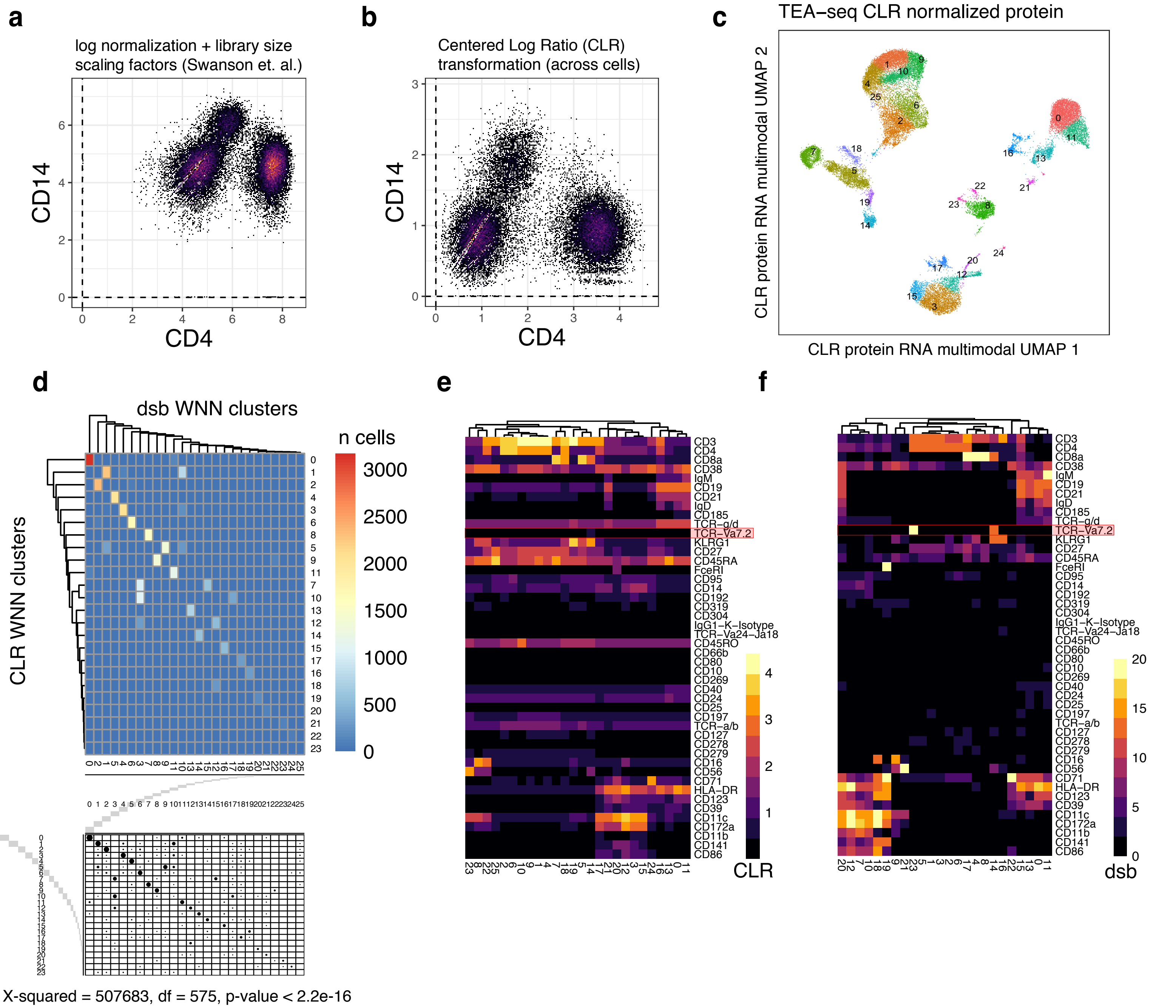
a
log normalization + library size scaling factors (Swanson et. al.)
CD14
CD4
b
Centered Log Ratio (CLR) transformation (across cells)
CD14
CD4
c
TEA−seq CLR normalized protein
CLR protein RNA multimodal UMAP 2
CLR protein RNA multimodal UMAP 1
d
dsb WNN clusters
CLR WNN clusters
n cells
3000
2500
2000
1500
1000
500
0
X-squared = 507683, df = 575, p-value < 2.2e-16
e
CLR
f
dsb
CD3
CD4
CD8a
CD38
IgM
CD19
CD21
IgD
CD185
TCR−g/d
TCR−Va7.2
KLRG1
CD27
CD45RA
FceRI
CD95
CD14
CD192
CD319
CD304
IgG1−K−Isotype
TCR−Va24−Ja18
CD45RO
CD66b
CD80
CD10
CD269
CD40
CD24
CD25
CD197
TCR−a/b
CD127
CD278
CD279
CD16
CD56
CD71
HLA−DR
CD123
CD39
CD11c
CD172a
CD11b
CD141
CD86

Supplementary Figure 9

Analysis of TEA-seq (transcriptome, epitopes and accessibility) tri-modal single cell assay data. **a.** TEA-seq data normalized by Library size based normalization (as in Swanson et. al.), and **b.** CLR across cells. **c.** UMAP plot of single cells and clusters derived by WNN joint mRNA-protein clustering with data normalized using CLR (see Fig. 4b for dsb normalized data). **d.** Contingency of clustering results between joint mRNA and protein Weighted Nearest Neighbor (WNN) clustering with CLR normalized (rows) or dsb normalized (columns) values as input to the protein matrix. The bottom margin shows the same data as circles with area proportional to frequency to show clusters with cell assignment differences. The average protein expression profiles of the clusters from **e.** CLR and **f.** dsb for protein normalization are shown as heatmaps.
