# Supplementary material for: Normalizing and denoising protein expression data from droplet-based single cell profiling
Source: Nat Commun. 2022 Apr 19;13:2099. doi: 10.1038/s41467-022-29356-8 (PMC9018908; doi:10.1038/s41467-022-29356-8)
Supplement: Supplementary file 3 — Description of Additional Supplementary Files [file 41467_2022_29356_MOESM3_ESM.pdf]

Description of Additional Supplementary Files

File Name: Supplementary Data 1
Description: Oligo-labelled antibodies from Biolegend used for cell hashing and surface target protein detection in the CITE-seq experiment
