# Supplementary material for: Normalizing and denoising protein expression data from droplet-based single cell profiling
Source: Nat Commun. 2022 Apr 19;13:2099. doi: 10.1038/s41467-022-29356-8 (PMC9018908; doi:10.1038/s41467-022-29356-8)
Supplement: Supplementary file 5 — Reporting Summary [file 41467_2022_29356_MOESM5_ESM.pdf]

nature research

Corresponding author(s): John S. Tsang

Last updated by author(s): Dec 2, 2021

Reporting Summary

Nature Research wishes to improve the reproducibility of the work that we publish. This form provides structure for consistency and transparency in reporting. For further information on Nature Research policies, see our Editorial Policies and the Editorial Policy Checklist.

Statistics

For all statistical analyses, confirm that the following items are present in the figure legend, table legend, main text, or Methods section.

n/a	Confirmed	
☐	☒	The exact sample size (*n*) for each experimental group/condition, given as a discrete number and unit of measurement
☐	☒	A statement on whether measurements were taken from distinct samples or whether the same sample was measured repeatedly
☐	☒	The statistical test(s) used AND whether they are one- or two-sided<br>*Only common tests should be described solely by name; describe more complex techniques in the Methods section.*
☐	☒	A description of all covariates tested
☐	☒	A description of any assumptions or corrections, such as tests of normality and adjustment for multiple comparisons
☐	☒	A full description of the statistical parameters including central tendency (e.g. means) or other basic estimates (e.g. regression coefficient) AND variation (e.g. standard deviation) or associated estimates of uncertainty (e.g. confidence intervals)
☐	☒	For null hypothesis testing, the test statistic (e.g. *F*, *t*, *r*) with confidence intervals, effect sizes, degrees of freedom and *P* value noted<br>*Give P values as exact values whenever suitable.*
☒	☐	For Bayesian analysis, information on the choice of priors and Markov chain Monte Carlo settings
☒	☐	For hierarchical and complex designs, identification of the appropriate level for tests and full reporting of outcomes
☐	☒	Estimates of effect sizes (e.g. Cohen's *d*, Pearson's *r*), indicating how they were calculated

Our web collection on statistics for biologists contains articles on many of the points above.

Software and code

Policy information about availability of computer code

Data collection	GEOquery_2.58.0 in R v4.0.5 was used for downloading TEA-seq metadata. All other data analyzed was publicly available and was downloaded from GEO or github repositories manually (see Data section below).
Data analysis	The software this manuscript describes, dsb, is available on CRAN:<br>https://cran.r-project.org/package=dsb<br><br>Two versions of R software v3.5.3 and v4.0.5 were used for the analysis in this manuscript, each includes separate package dependency versions. The main software package versions that correspond to each version of R include (format = package_version):<br>R v3.5.3: mclust_5.4.5, limma_3.38.3, Seurat_2.3.4, tidyverse_1.2.1, broom_0.5.2, reticulate_1.12, umap_0.2.3.1, here_0.1<br>R v4.0.5: mclust_5.4.7, limma_3.46.0, Seurat_4.0.1, tidyverse_1.3.0, broom_0.7.5, here_1.0.1<br><br>A list of all R software package versions that run with either of the 2 versions of R with installation instructions are listed in the reproducible workflow:<br>https://github.com/niaid/dsb_manuscript<br>In addition, all additional package dependencies with version number are reproduced at the end of each .r analysis script in the reproducible analysis code above as commented sessionInfo() output.<br><br>Software used for low-level processing of the CITE-seq data was previously reported in our prior manuscript with the initial report of the PBMC CITE-seq dataset used to develop dsb: https://www.nature.com/articles/s41591-020-0769-8<br>The main software packages for low level processing included:<br>Bcl2fastq version 2.20 (Illumina)<br>CellRanger version 3.0.1 (10x Genomics)

CITE-seqCount version 1.4.2

For manuscripts utilizing custom algorithms or software that are central to the research but not yet described in published literature, software must be made available to editors and reviewers. We strongly encourage code deposition in a community repository (e.g. GitHub). See the Nature Research guidelines for submitting code & software for further information.

Data

Policy information about availability of data

All manuscripts must include a data availability statement. This statement should provide the following information, where applicable:

- Accession codes, unique identifiers, or web links for publicly available datasets
- A list of figures that have associated raw data
- A description of any restrictions on data availability

Data used in this study are available to download in the data repository associated with this manuscript:
https://doi.org/10.35092/yhjc.13370915.
Instructions for using the data to reproduce analysis reported in this manuscript are available in the reproducible analysis workflow:
https://github.com/niaid/dsb_manuscript/.
The public datasets included in the data repository were downloaded online and are also available from 10X genomics at:
https://support.10xgenomics.com single-cell-gene-expression/datasets
and from Mission Bio at:
https://missionbio.com/capabilities/dna-protein/#Data.
ASAP-seq data was downloaded from GEO with accession number GSE156477:
https://www.ncbi.nlm.nih.gov/geo/query/acc.cgi?acc=GSE156477
and from the repository:
https://github.com/caleblareau/asap_reproducibility
TEA-seq data was downloaded from GEO with accession number GSE158013:
https://www.ncbi.nlm.nih.gov/geo/query/acc.cgi?acc=GSE158013

Field-specific reporting

Please select the one below that is the best fit for your research. If you are not sure, read the appropriate sections before making your selection.

☒ Life sciences ☐ Behavioural & social sciences ☐ Ecological, evolutionary & environmental sciences

For a reference copy of the document with all sections, see nature.com/documents/nr-reporting-summary-flat.pdf

Life sciences study design

All studies must disclose on these points even when the disclosure is negative.

Sample size	This manuscript describes a method for low level denoising and normalization of protein UMI count data derived from a CITE-seq experiment with >50,000 single cells which is then further validated on additional datasets ranging from <2000 cells to ~30,000 cells. Sample groups are not compared in these normalization analysis. Sample size calculations were not performed a priori in this methodology development manuscript, where comparisons were between thousands of cells per comparison. This provides sufficient n for statistical comparisons.
Data exclusions	No data were excluded from analysis, some data points were excluded from visualizations to focus on main populations of cells as noted in figure legends and in the text.
Replication	The method was tested on 8 datasets from different single cell assay technologies. All replication attempts were successful as detailed in the text and summarized in Figure 2i.
Randomization	There were no variables or interventions to randomize in this study.
Blinding	Blinding is not relevant to this method development study, investigators could not be blinded during data collection or analysis because there was not an experimental perturbation / intervention being measured.

Reporting for specific materials, systems and methods

We require information from authors about some types of materials, experimental systems and methods used in many studies. Here, indicate whether each material, system or method listed is relevant to your study. If you are not sure if a list item applies to your research, read the appropriate section before selecting a response.

Materials & experimental systems

n/a	Involved in the study
☐	☒ Antibodies
☒	☐ Eukaryotic cell lines
☒	☐ Palaeontology and archaeology
☒	☐ Animals and other organisms
☐	☒ Human research participants
☒	☐ Clinical data
☒	☐ Dual use research of concern

Methods

n/a	Involved in the study
☒	☐ ChIP-seq
☒	☐ Flow cytometry
☒	☐ MRI-based neuroimaging

Antibodies

Antibodies used

Antibodies are reported in the Reporting Summary of our initial report of this dataset:
https://www.nature.com/articles/s41591-020-0769-8 DOI: 10.1038/s41591-020-0769-8
The table is reproduced here in Supplementary Table 1.

Validation

Quality control and reproducibility statements are available on the following Biolegend TotalSeq-A website:
https://www.biolegend.com/en-us/quality-control
https://www.biolegend.com/en-us/reproducibility

Human research participants

Policy information about studies involving human research participants

Population characteristics

PBMC samples for CITE-seq were acquired previously (see Tsang et al, Cell 2014 DOI: 10.1016/j.cell.2014.03.031). Subject level data are not analyzed in this low level normalization method paper but are available in the manuscript above.

Recruitment

PBMC used for CITE-seq were collected prior to the study (see Tsang et al, Cell 2014 DOI: 10.1016/j.cell.2014.03.031).

Ethics oversight

The study protocol was approved by the NIH Institutional Review Board (09-H-0239)

Note that full information on the approval of the study protocol must also be provided in the manuscript.
